# Integrated bioinformatical analysis, machine learning and *in vitro* experiment-identified m6A subtype, and predictive drug target signatures for diagnosing renal fibrosis

**DOI:** 10.3389/fphar.2022.909784

**Published:** 2022-08-31

**Authors:** Chunxiang Feng, Zhixian Wang, Chang Liu, Shiliang Liu, Yuxi Wang, Yuanyuan Zeng, Qianqian Wang, Tianming Peng, Xiaoyong Pu, Jiumin Liu

**Affiliations:** ^1^ Department of Urology, Guangdong Provincial People’s Hospital, Guangdong Academy of Medical Sciences, Guangdong Guangzhou, Wuhan, China; ^2^ Department of Urology, Wuhan Hospital of Traditional Chinese and Western Medicine, Tongji Medical College, Huazhong University of Science and Technology, Wuhan, China; ^3^ Department of Urology, Wuhan No. 1 Hospital, Wuhan, China; ^4^ Department of Geriatrics, Tongji Hospital, Tongji Medical College, Huazhong University of Science and Technology, Wuhan, China; ^5^ Department of Urology, Tongji Hospital, Tongji Medical College, Huazhong University of Science and Technology, Wuhan, China; ^6^ Department of Nephrology, Tongji Hospital, Tongji Medical College, Huazhong University of Science and Technology, Wuhan, China; ^7^ School of Life Science and Engineering, Southwest Jiaotong University, Chengdu, China

**Keywords:** logistic regression, prective model, drug sensitivity, renal fibrosis, immune microenvironment

## Abstract

Renal biopsy is the gold standard for defining renal fibrosis which causes calcium deposits in the kidneys. Persistent calcium deposition leads to kidney inflammation, cell necrosis, and is related to serious kidney diseases. However, it is invasive and involves the risk of complications such as bleeding, especially in patients with end-stage renal diseases. Therefore, it is necessary to identify specific diagnostic biomarkers for renal fibrosis. This study aimed to develop a predictive drug target signature to diagnose renal fibrosis based on m6A subtypes. We then performed an unsupervised consensus clustering analysis to identify three different m6A subtypes of renal fibrosis based on the expressions of 21 m6A regulators. We evaluated the immune infiltration characteristics and expression of canonical immune checkpoints and immune-related genes with distinct m6A modification patterns. Subsequently, we performed the WGCNA analysis using the expression data of 1,611 drug targets to identify 474 genes associated with the m6A modification. 92 overlapping drug targets between WGCNA and DEGs (renal fibrosis vs. normal samples) were defined as key drug targets. A five target gene predictive model was developed through the combination of LASSO regression and stepwise logistic regression (LASSO-SLR) to diagnose renal fibrosis. We further performed drug sensitivity analysis and extracellular matrix analysis on model genes. The ROC curve showed that the risk score (AUC = 0.863) performed well in diagnosing renal fibrosis in the training dataset. In addition, the external validation dataset further confirmed the outstanding predictive performance of the risk score (AUC = 0.755). These results indicate that the risk model has an excellent predictive performance for diagnosing the disease. Furthermore, our results show that this 5-target gene model is significantly associated with many drugs and extracellular matrix activities. Finally, the expression levels of both predictive signature genes EGR1 and PLA2G4A were validated in renal fibrosis and adjacent normal tissues by using qRT-PCR and Western blot method.

## Introduction

Renal fibrosis is a process of wound-healing failure in renal tissues after chronic injury, calcium deposits, and inflammation. It is a common pathway and pathological marker of almost all types of chronic kidney diseases (CKD) (Farup et al., 2021) (Xie et al., 2021). The main pathological feature of renal fibrosis is nephrogenesis. Fibroblasts are massively activated and proliferated. The extracellular matrix (ECM) deposited in the renal interstitium is excessively synthesized and secreted, resulting in structural damage, renal function impairment, and, ultimately end-stage renal disease (Yan et al., 2021). Renal biopsy is the gold standard for defining renal fibrosis. However, it is invasive and involves the risk of complications such as bleeding, especially in patients with end-stage renal diseases. Therefore, it is necessary to search for more accessible and specific biomarkers of renal fibrosis (Jia et al., 2020).

Despite the significant progress made by modern medicine, non-invasive diagnostic techniques (Nielsen et al., 2020) and effective treatment measures for renal fibrosis are still limited (Zhang et al., 2021a). Currently, there is no specific treatment (Deng et al., 2020). In recent years, many efforts have been devoted to finding novel biomarkers and therapeutics of renal fibrosis, focusing on the properties of unknown mediators and pathways involved in developing renal fibrosis (Prakoura et al., 2019). Machine learning (ML) is an emerging field with enormous resources being applied to medical problems that fuse computer science and statistics together (Handelman et al., 2018). So far, it has shown good performance in a wide range of tasks in biomedicines (Cilluffo et al., 2021). Machine learning techniques have been widely applied to identify disease biomarkers for diagnosis, prognosis, and risk assessment (Dockès et al., 2021; Field et al., 2021).

Combined with machine learning, Cao et al. (2016) found that the urinary TREM-1/TREM-2 ratio can be a potential biomarker for diagnosing renal fibrosis in CKD patients (Guerquin et al., 2013). Hypoxia promotes the development of renal fibrosis. Armutcu ADAMTS protease may provide some important signals for the early diagnosis and treatment of renal fibrosis. Elevated plasma CDH11, SMOC2, and PEDF and urinary CDH11 and PEDF levels were associated with interstitial fibrosis and significantly correlated with increased severity of tubular atrophy. In both the cohorts, elevated plasma and urinary SMOC2 and urinary CDH11 levels were independently associated with progression to end-stage renal diseases (Ma et al., 2021a). These biomarkers provided new ideas for treating renal fibrosis. EVR may delay impaired autophagic flux and block the activation of the NF-kB pathway (Wang et al., 2019), and rAAV9 acts as a carrier of miR-29b anti-fibrotic factors (Xu et al., 2020). Twist1/galectin-3 signaling pathway regulates macrophage plasticity (M2 phenotype) and promotes renal fibrosis (Liu et al., 2021). However, the underlying mechanisms of renal fibrosis have not been fully elucidated, and current treatments can only delay disease progression. Therefore, exploring novel potential drug targets are of great significance for the treatment of renal fibrosis (Sun et al., 2022).

Massive gene expression profiling databases provide opportunities to discover novel prognostic and predictive biomarkers using sophisticated deep learning algorithms. These datasets also allow extensive external validation (Sun et al., 2022). We first searched three datasets from the GEO database, removed the batch correspondence through the SVA algorithm, and merged them into the training dataset. We then performed a panel of 21 putative m6A regulators (7 writers, 12 readers and 2 erasers) to identify distinct patterns of m6A methylation modification. We then performed an unsupervised consensus clustering analysis of 175 renal fibrosis samples based on the expressions of 21 m6A regulators. To further clarify the role of m6A modification patterns in the immune microenvironment, we used the ESTIMATE algorithm to evaluate immune infiltration characteristics in different m6A patterns. We next analyzed the expression of canonical immune checkpoints and immune-related genes with distinct m6A modification patterns. We then performed the WGCNA analysis using the expression data of 1,611 drug targets obtained from the GeneCards database to identify 474 genes associated with m6A modification. 92 overlapping drug targets between WGCNA and DEGs (renal fibrosis vs. normal samples) were defined as key drug targets. A prediction model for renal fibrosis composed of five drug targets was developed using LASSO regression and stepwise logistic regression (LASSO-LR). The ROC curve shows that the risk model has an excellent predictive performance. Finally, we performed drug sensitivity and gene and extracellular matrix analysis. Five genes were significantly associated with drug sensitivity and extracellular matrix activity.

## Materials and methods

### RNA-sequencing data and data preprocessing

The expression profile dataset was searched from the NCBI GEO database (http://www.ncbi.nlm.nih.gov/geo/) with “Renal fibrosis, *Homo sapiens*” as keywords, and a total of three sets of expression data. The analysis of the m6A modulators was performed after batch effects were removed by the SVA algorithm.

### Identification of m6A-modified subtypes

An unsupervised consensus clustering analysis was performed on 175 renal fibrosis samples based on the expressions of 21 m6A regulators. The principal component analysis (PCA) showed that the three subtypes could clearly distinguish the samples. We performed the GSVA analysis using the R package. The GSVA algorithm calculated the m6A score between the three subtypes. Significant differences in the m6A score were found among the three subtypes.

### Immune infiltration characterization

We obtained microenvironment scores through the ESTIMATE package to quantify the immune microenvironment (TME) levels across different m6A patterns. We further employed the CIBERSORT method to compare the infiltration levels of 22 immune cells among the three m6A subtypes. Finally, we performed a GSVA enrichment analysis of the enrichment scores for the immune gene set using the R package. The results showed significant differences in the enrichment of 17 immune gene sets among the three m6A subtypes. Adjusted *p* < 0.05 was considered statistically significant.

### Detection of m6A modification-related drug target modules based on WGCNA

WCGNA clustered drug targets with similar expression patterns to construct a scale-free gene co-expression network and analyzed the correlation between modules and specific phenotypes (m6A modified subtypes). According to the correlation between modules and target genes and the correlation between modules, important target gene modules are screened. It has significant advantages in analyzing gene association patterns as a comprehensive biological algorithm. Two of its highlights are clustering modules with genes with similar expression patterns and the correlation analysis between modules and m6A-modified subtypes. This study performed a hierarchical cluster analysis on the expression profiles to exclude outliers. Subsequently, the Pearson correlation coefficient of any two genes was calculated, and a correlation matrix was established. The topological overlap matrix was transformed into a topological overlap matrix using the topological overlap matrix similarity function. Co-expressed genes were assigned into modules by a dynamic minimal tree-cutting algorithm. The module genes with the highest correlation were obtained for subsequent analysis.

### Differential identification and functional enrichment analysis

DAVID is an online bioinformatics tool designed to predict many gene functions. Therefore, we used DAVID to observe the gene enrichment of the pathways (*p* < 0.05). We used the clusterProfiler R package to perform a functional annotation of the key genes.

### Least absolute contraction and selection operator analysis

Drug targets associated with renal fibrosis were integrated in least absolute contraction and selection operator (LASSO) regression to identify prognostic biomarkers. We use the “timeeroc” package to plot the receiver operator characteristic (ROC) curves of the datasets separately. Then, we calculated and compared the area under the curve (AUC) of the ROC curves to test the performance of the classifier.

### qRT-PCR

Total RNA was extracted by the TRIzol reagent. Then, qRT-PCR was performed with One Plus Step (Thermo Fisher, United States) and SYBR Premix (Takara, Japan) following the manufacturer’s instructions. The primer sequences of the relevant genes are listed in Supplementary Table S1. A unilateral ureteral obstruction (UUO) kidney disease model was established for harvesting fibrotic kidneys. C57Bl/6J mice (SLAC Laboratory Animal Company) were given left ureteral ligation to establish a UUO model as previously described (Guerquin et al., 2013).

### Western blotting

To detect the EGR1 and PLA2G4A protein expression levels, the protein was extracted by a radio-immunoprecipitation assay protein lysis buffer (Beyotime Institute of Biotechnology) and FOCUS Global Fractionation kit (G-Biosciences). The proteins were separated by SDS-PAGE and transferred onto polyvinyl difluoride membranes. The membranes were incubated in 5 % non-fat milk for blocking, followed by incubation with primary antibodies EGR1 and PLA2G4A (1:1,000, Abclonal Technology) at 4°C overnight. Finally, the membranes were washed and incubated in a blocking buffer with horseradish peroxidase-conjugated secondary antibodies for 2 h before detection.

## Results

### Data collection and de-batch processing

Expression profile datasets were searched from the NCBI GEO database (http://www.ncbi.nlm.nih.gov/geo/; March 2022) with “Renal fibrosis, *Homo sapiens*” as keywords. A total of three sets of the expression data were obtained:

A. GSE22459: 65 samples; 25 normal and 40 renal fibrosis samples were included. Sequencing platform: GPL570.
B. GSE76882: Contains 234 samples; 99 normal and 135 renal fibrosis samples. Sequencing platform: GPL13158.
C. GSE57731: Contains 73 samples; 45 normal and 28 renal fibrosis samples. Sequencing platform: GPL17244 for the subsequent validation analysis.

**FIGURE 1:**
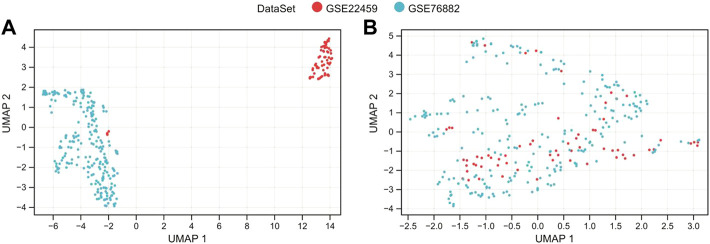
De-batch analysis. **(A,B)** are the sample step-by-step diagrams before and after de-batching, respectively.

Then, the SVA algorithm removed the two sets of the gene expression profile data of A and B and merged them into a training dataset containing 124 normal and 175 renal fibrosis samples. The sample relationship distribution before and after batch effect removal is shown in Figures 1A, B.

### Expression landscape of m6A regulators

**FIGURE 2:**
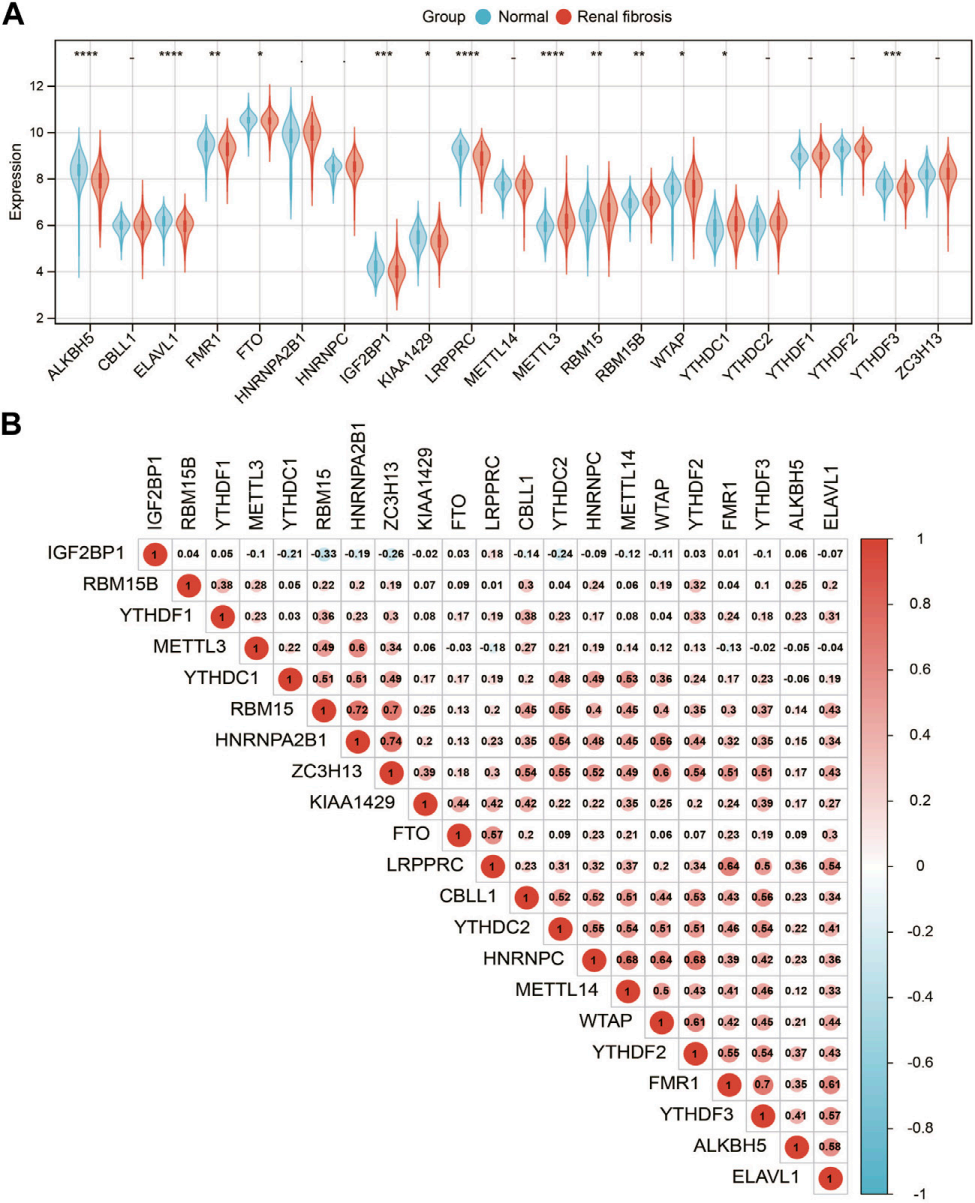
Expression landscape of m6A RNA methylation regulators in renal fibrosis. **(A)** Boxplot of the transcriptome expressions of 21 m6A regulators between healthy and renal fibrosis samples. **(B)** Heatmap of expression level correlations of 21 m6A regulators. * means *p* less than 0.05; ** means *p* less than 0.01, *** means *p* less than 0.001.

We analyzed a panel of 21 putative m6A regulators (7 writers, 12 readers, and 2 erasers) to identify distinct patterns of m6A methylation modification. As shown in Figures 2A 13 m6A regulators were significantly different between renal fibrosis and normal samples (*p* < 0.05). To explore the association between different m6A modulators, we describe the correlation pattern between three m6A modulators (Figure 2B).

### Identification of m6A modification subtypes in renal fibrosis

**FIGURE 3:**
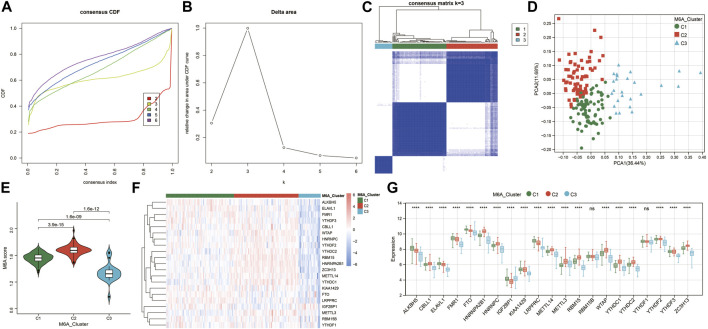
Identification of m6A-modified subtypes. **(A)** Consensus cluster cumulative distribution function (CDF) for *k* = 2–6. **(B)** Relative change in the area under the CDF curve at *k* = 2–6. **(C)** Consensus clustering matrix for optimal *k* = 3. **(D)** Principal component analysis (PCA) of the three m6A subtypes in renal fibrosis. **(E)** Violin plot of m6A score differences among the three m6A subtypes. **(F)** Heatmap of the m6A regulator expression among the three m6A subtypes. Red represents high expression and blue represents low expression. **(G)** The expression statuses of 21 m6A regulators in the three m6A subtypes. * means *p* less than 0.05; ** means *p* less than 0.01, *** means *p* less than 0.001.

To investigate the m6A modification patterns in renal fibrosis, we performed an unsupervised consensus clustering analysis of 175 renal fibrosis samples based on the expressions of 21 m6A regulators (Figures 3A–C). By setting the K value in the range of 2–6 and choosing the optimal K = 3, three different m6A modified subtypes of renal fibrosis were identified, among which, C1, C2, and C3 contained 77, 73, and 25 samples, respectively. The principal component analysis (PCA) showed that the three subtypes could clearly distinguish the samples (Figure 3D). We calculated m6A scores by using the GSVA algorithm and found that m6A scores were significantly different among the three subtypes (Figure 3E). There are diverse m6A modification patterns. In addition, we also found 19 m6A regulators whose expressions were significantly different among the three subtypes (Figures 3F,G). Notably, except for IGF2BP1, the remaining m6A regulators generated a unique m6A low transcription profile in C3.

### Characteristics of immune infiltration in different m6A patterns

**FIGURE 4:**
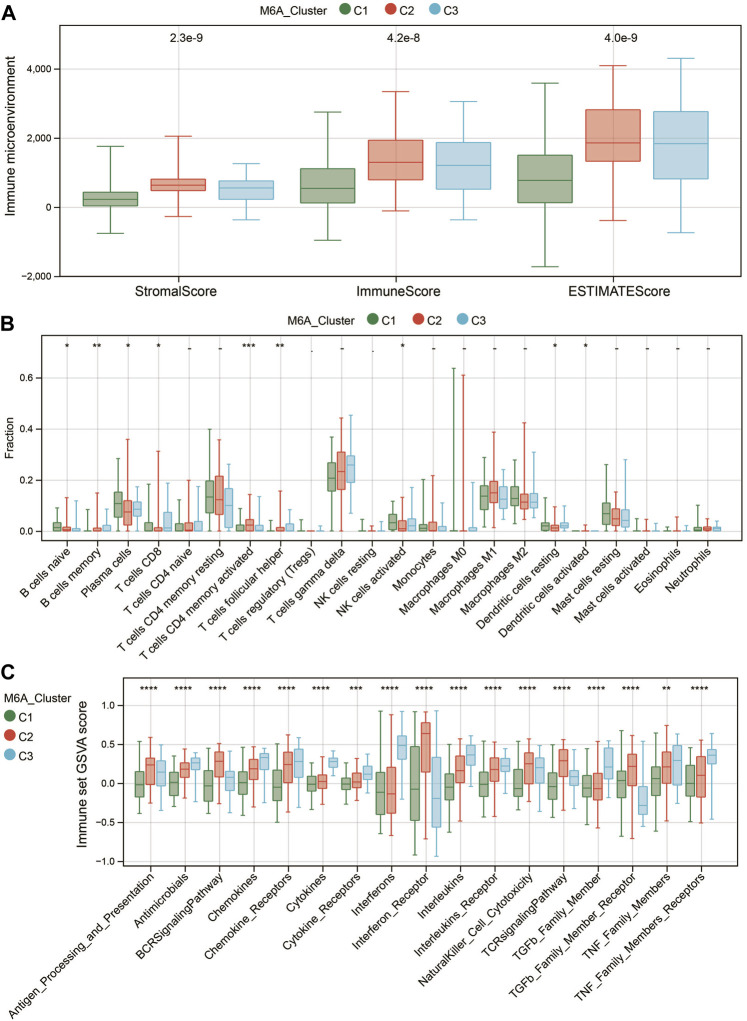
Characteristics of the immune microenvironment in different m6A modification patterns. **(A)** Differences in TME scores among the three m6A-modified subtypes. **(B)** Differences in the abundance of 22 immune cells among the three m6A modification patterns. **(C)** Differences in the activity of 17 immune response gene sets under the three m6A modification patterns. * means *p* less than 0.05; ** means *p* less than 0.01, *** means *p* less than 0.001.

Recent studies have shown that the m6A modification of RNA plays a crucial role in forming immune responses and the immune environment. Then, we utilized the ESTIMATE algorithm to measure the immune microenvironment (TME) level. C2 exhibited higher immune and stromal scores (Figure 4A). We further compared the infiltration levels of 22 immune cells among the three m6A subtypes using the CIBERSORT method (Figure 4B). Nine significantly differentially expressed immune cells (DICs) were found (*p* < 0.05). To better illustrate the immune gene set activity changes among m6A modified subtypes, we calculated the enrichment scores for 17 immune gene sets from the ImmPort database using GSVA. The results showed significant differences in the enrichment of the 17 immune gene sets among the three m6A subtypes (Figure 4C). These results demonstrated that m6A methylation modification had an essential regulatory role in shaping different immune microenvironments in renal fibrosis.

### Immune checkpoints, MHC, co-suppression, and co-stimulatory molecular signatures in different m6A patterns

**FIGURE 5:**
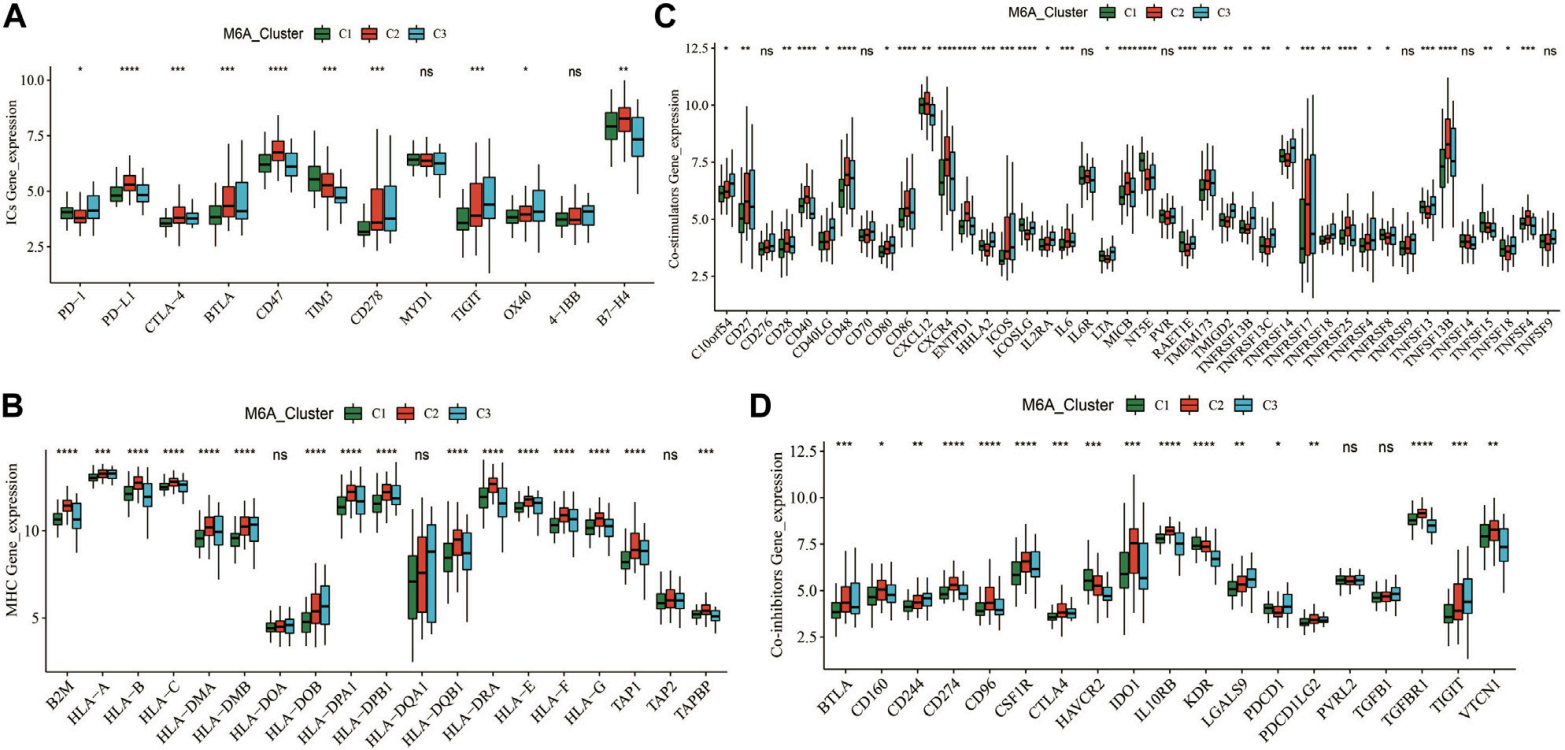
Expression patterns of typical immune-related genes. **(A–D)** Differential expression of immune checkpoint, MHC, co-suppression and co-stimulatory genes among different m6A clusters. * means *p* less than 0.05; ** means *p* less than 0.01, *** means *p* less than 0.001.

We further analyzed the expression of canonical immune checkpoints and immune-related genes with different m6A modification patterns. The expressions of PD-1, PD-L1, CTLA-4, BTLA, CD47, TIM3, CD278, TIGIT, OX40, and B7-H4 were significantly different among the three subtypes (Figure 5A). In addition, the expressions of multiple MHC, co-stimulatory, and co-repressor-related genes were significantly different across m6A (Figures 5B–D). This means that the m6A modification patterns may benefit more from immunotherapy responses.

### WGCNA analysis revealed m6A modification-related drug targets

**FIGURE 6:**
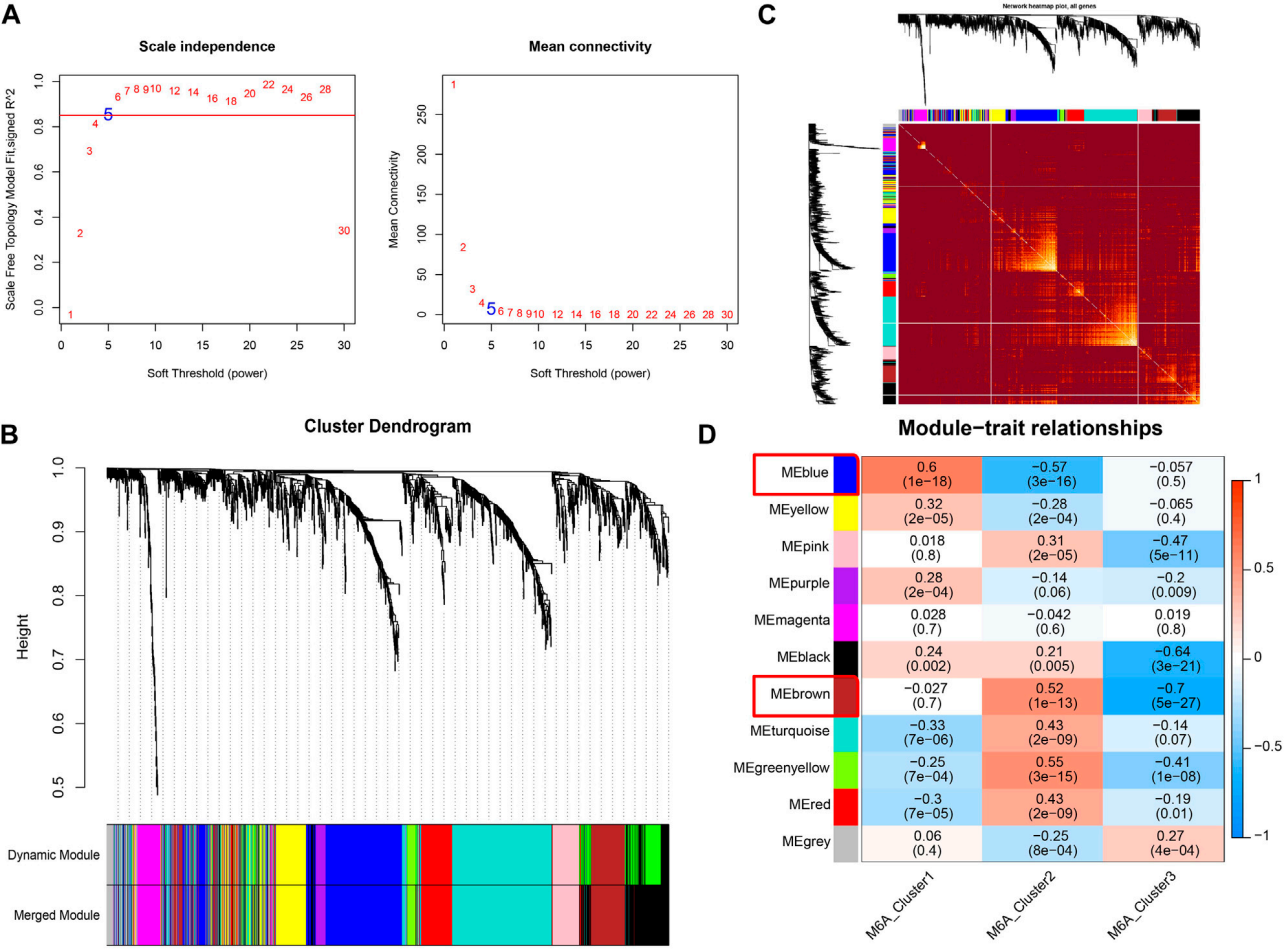
Co-expression identifies drug targets associated with m6A modification. **(A)** Left image: Adjacency matrix weight parameter power selection graph. The horizontal axis represents the weight parameter power, and the vertical axis represents the square of the correlation coefficient between log (k) and log [p(k)] in the corresponding network. The higher the squared value of the correlation coefficient, the closer the network is to the distribution without network scale. The red line represents the standard line where the squared value of the correlation coefficient reaches 0.85. Right image: Schematic diagram of the average connection degree of genes under different adjacency matrix weight parameters, power parameter. The red line represents the value of the average connection degree of network nodes under the value of the power parameter of the adjacency matrix weight parameter in the left image. **(B)** Module division tree diagram. Each color represents a different module. **(C)** Heatmap of the Topological Overlap Metric (TOM) matrix. Light yellow represents lower TOM, and darker red represents higher TOM. **(D)** Heatmap of the correlations of individual modules with m6A-modified subtypes.

The m6A modification subtype was used as the clinical feature to implement the WGCNA analysis. The expression data of 1,611 drug targets that were obtained from the GeneCards database (relevance score >7) were used. We set the network construction parameter and calculated the scale-free distribution topology matrix. As shown in Figure 6A, we selected the power five when the squared value of the correlation coefficient reached 0.85 for the first time (red line). The 1,611 drug targets were divided into 11 modules based on dynamic pruning (Figure 6B) and topological overlap measurement (TOM) clustering (Figure 6C). Subsequently, the associations between each module and clinical features were calculated (Figure 6D). We selected the blue and brown modules with the highest degree of correlation with m6A modified subtypes. Therefore, the 474 genes in blue and brown modules served as m6A modification-related drug targets for the subsequent analysis.

### Differential gene identification and functional enrichment analysis

**FIGURE 7:**
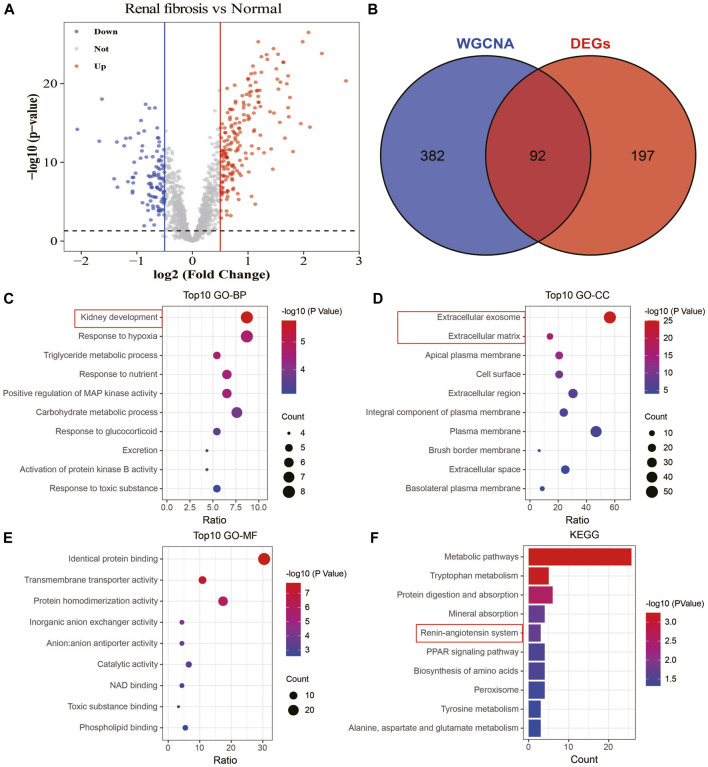
Identification and enrichment analysis of key drug targets. **(A)** Volcano plot showing information on changes in the expressions of drug targets between healthy and renal fibrosis samples. **(B)** Venn plot of the intersection of WGCNA and differential analysis. **(C)** Biological processes of the key drug targets. **(D)** Cellular components involved in the key drug targets. **(E)** Molecular functions of the key drug targets. **(F)** Signaling pathways involved in the key drug targets.

To understand the association between drug targets and renal fibrosis, we performed a differential gene analysis on renal fibrosis and normal samples based on the expression of 1,611 drug targets (FDR<0.05). 289 drug targets with altered expressions were identified (|log2FC|>0.5; Figure 7A). 92 overlapping drug targets between WGCNA and DEGs were defined as key drug targets (Figure 7B). To characterize the roles and underlying mechanisms of key genes, we performed Gene Ontology (GO) and KEGG analyses. The analyses showed that key genes were mainly related to kidney development, extracellular exosome, extracellular matrix, and the angiotensin system (Figures 7C–F). These findings suggest that key drug targets may be involved in the development and progression of renal fibrosis.

### Construction and validation of a risk model

**FIGURE 8:**
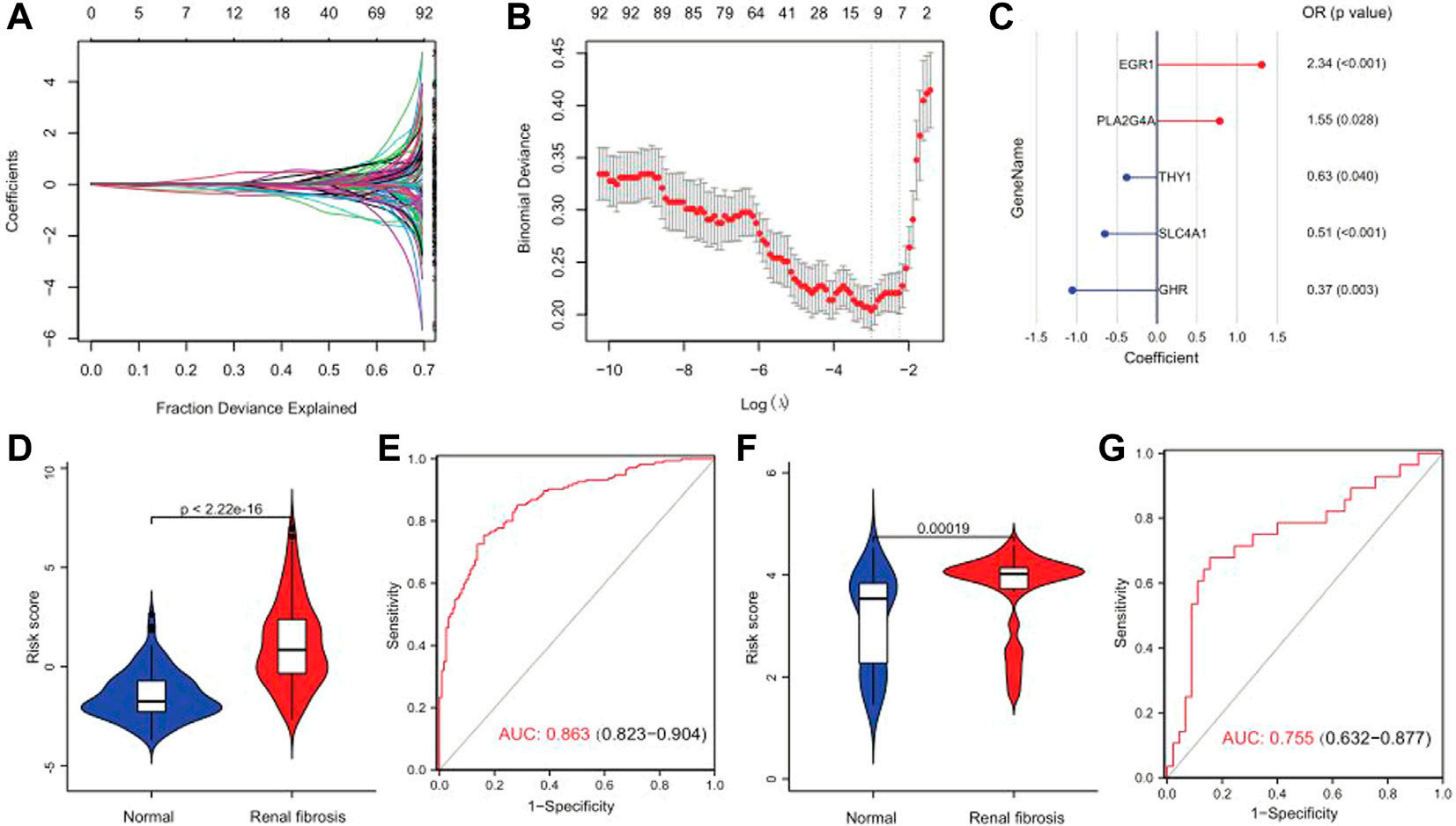
Risk scores can differentiate healthy and renal fibrosis samples. **(A)** Distribution of the LASSO coefficients for 92 drug targets. **(B)** 10-fold cross-validation with adjusted parameter selection in LASSO regression. Partial likelihood deviations are plotted against log (λ), where *λ* is the tuning parameter. **(C)** A gene signature with five drug targets was developed by stepwise logistic regression, and risk scores were calculated. **(D)** Violin plot of the risk score distribution between normal samples and renal fibrosis in the training set. **(E)** Predicted ROC curve for the risk scores in the training set. **(F)** Distribution of the differences in risk scores between normal samples and renal fibrosis in the GSE57731 validation set. **(G)** Predicted ROC curve of the GSE57731 validation central risk score.

A series of bioinformatics algorithms were used to further study the contribution of the key drug targets to the pathogenesis of renal fibrosis. First, we performed feature selection and dimensionality reduction on 92 drug targets related to renal fibrosis by using LASSO regression and found 11 important drug targets (Figures 8A,B). Subsequently, we developed a predictive model composed of five drug targets using stepwise logistic regression to diagnose renal fibrosis (Figure 8C). Finally, we calculated the risk score for each sample with the five drug target expression values and regression coefficients. The risk score is calculated as follows: RiskScore = 10 + (0.4402 × PLA2G4A) + (−0.4683 × THY1) + (0.8501 × EGR1) + (-0.9986 × EGR1) + (−0.6811 × SLC4A1). The risk score could differentiate between healthy and renal fibrosis samples well, with renal fibrosis having a much higher risk score than the healthy samples (Figure 8D). The ROC curve showed that the risk score (AUC = 0.863) performed well in predicting renal fibrosis (Figure 8E). In addition, the external validation set GSE57731 also further confirmed the predictive performance of the risk score (AUC = 0.755) (Figures 8F,G). Therefore, these results indicate that the risk model has excellent predictive performance.

### Drug sensitivity analysis

**FIGURE 9:**
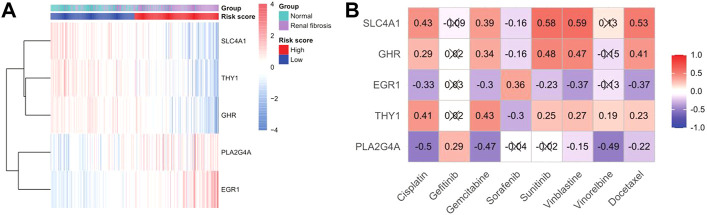
Sensitivity analysis of gene and drug therapy. **(A)** Heatmap of the expression of model genes in disease and risk groups. **(B)** Heatmap of model gene–drug associations.

We drew a heat map of the model genes to further explore the role of model genes in the development of renal fibrosis and the correlation between drug treatments (Figure 9A). We found that SLC4A1, THY1, and GHR were significantly under-expressed in renal fibrosis, and PLA2G4A and EGR1 were significantly over-expressed compared with the normal group. Subsequently, the sensitivity of each renal fibrosis patient to drug treatment was estimated based on the Genomics of Cancer Drug Sensitivity (GDSC) database. IC50 quantification was performed by the pRRophetic package in R. We compared the IC50 levels of five model genes with eight drugs (Figure 9B) and found that the IC50 levels of cisplatin, gemcitabine, vinblastine, and docetaxel significantly correlated with the expressions of the five model genes.

### Analysis of genes and extracellular matrix

**FIGURE 10:**
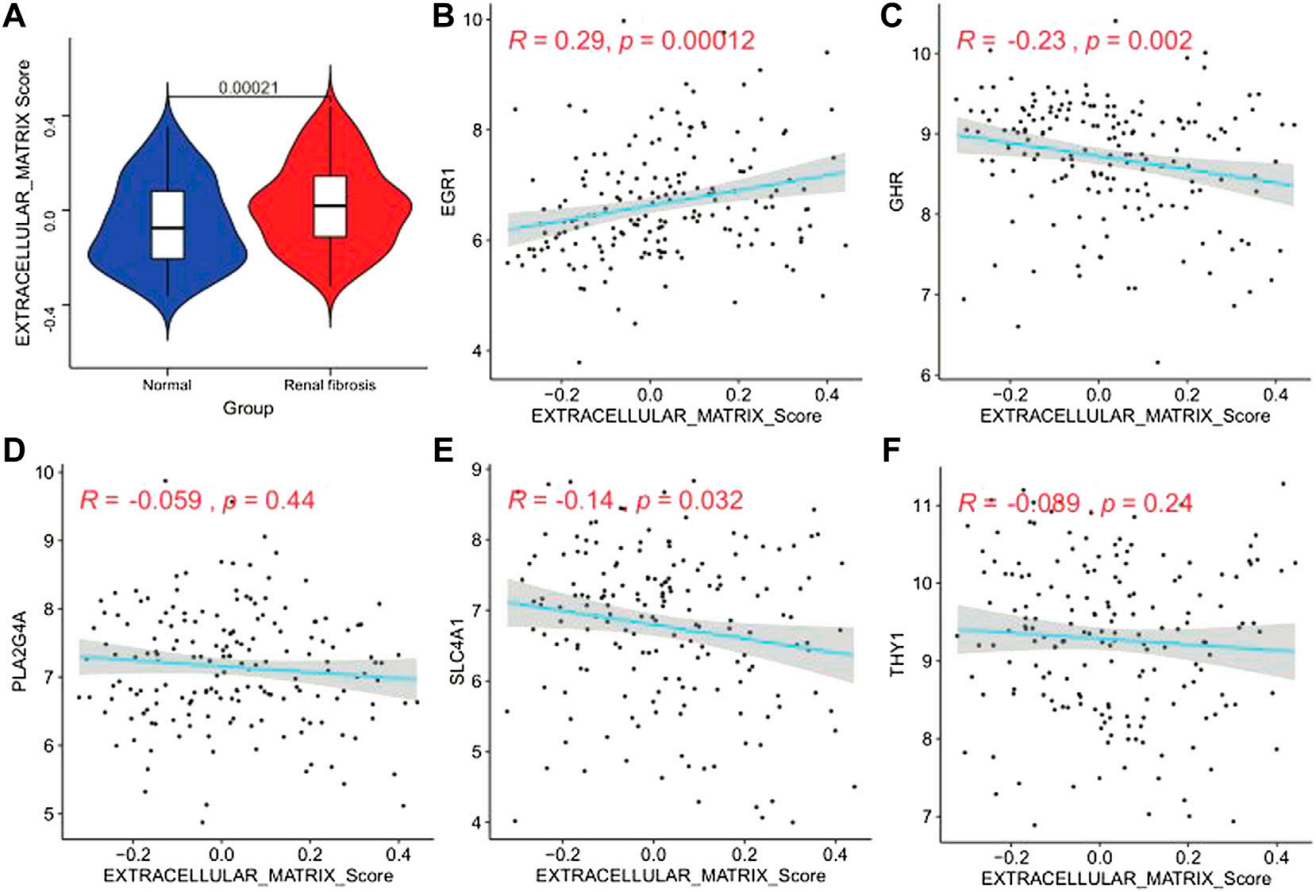
Extracellular matrix activity profile. **(A)** Differential distribution of extracellular matrix activity in normal and renal fibrotic tissues, **(B–F)** scatter of the correlations between five genes (EGR1, GHR, PLA2G4A, SLC4A1, and THY1) and extracellular matrix activity in renal fibrotic tissues.

Studies have found that renal fibrosis is significantly correlated with the extracellular matrix. Therefore, we used the GSVA enrichment analysis to evaluate the activation state of the extracellular matrix. The extracellular matrix enrichment in renal fibrosis scores was significantly higher than that of normal tissues (*p* = 0.00021; Figure 10A). We performed a correlation analysis to further explore the ECM activity status regulated by the five model genes (Figures 10B–F). We found that EGR1 was significantly positively correlated with ECM activity (*r* = 0.29, *p* = 0.00012). GHR (*r* = −0.23, *p* = 0.002) and SLC4A1 (*r* = −0.14, *p* = 0.032) were significantly negatively correlated with the extracellular matrix activity. PLA2G4A and THY1 did not correlate with the extracellular matrix activity.

### Validating the expression levels of EGR1 and PLA2G4A *via* qRT-PCR and Western blot

**FIGURE 11:**
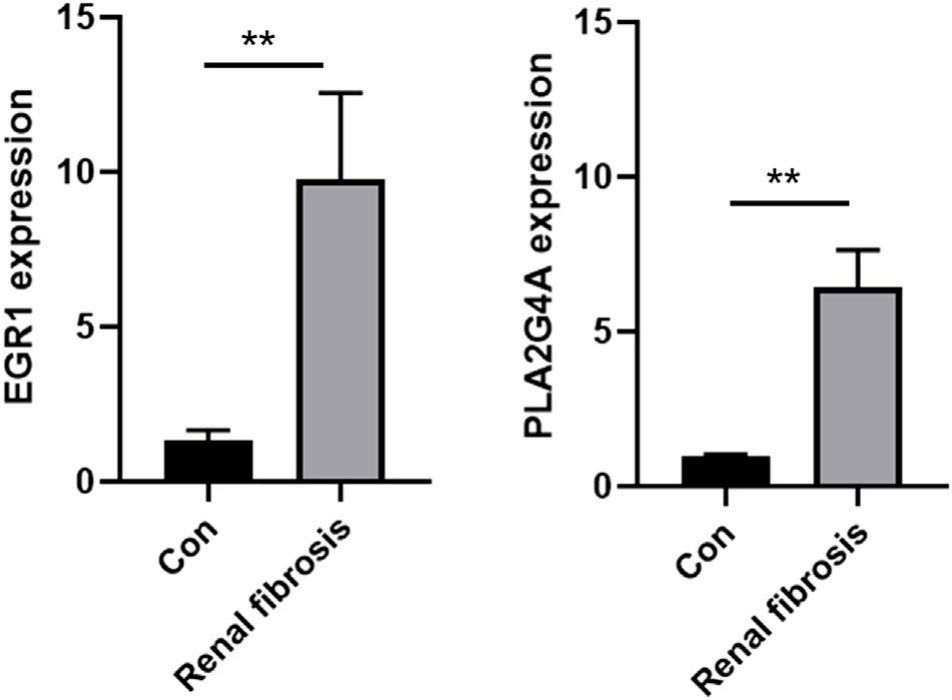
EGR1 and PLA2G4A mRNA expressions were analyzed by using the qRT-PCR assay in renal fibrosis and adjacent normal tissues.

**FIGURE 12:**
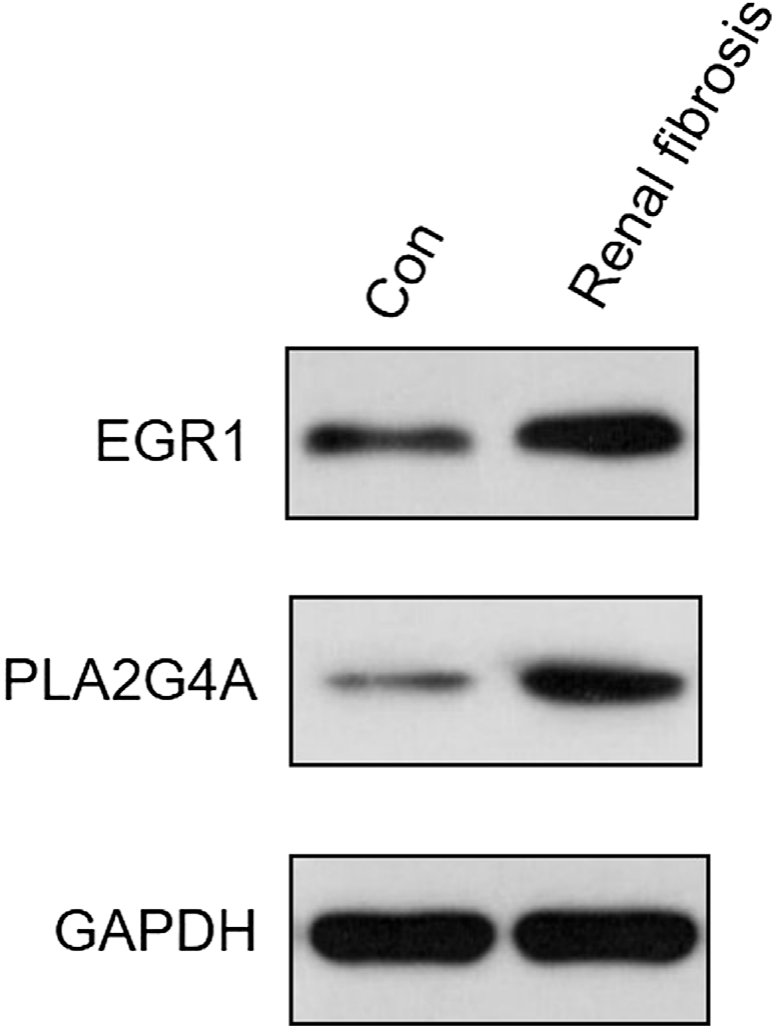
EGR1 and PLA2G4A protein expressions were analyzed by Western blot in renal fibrosis and adjacent normal tissues.

The expression levels of both EGR1 and PLA2G4A were detected in renal fibrosis and adjacent normal tissues by qRT-PCR and Western blot method. Our results revealed that, compared with normal tissues, the EGR1 and PLA2G4A genes have higher expressions in tissues of renal fibrosis (*p* < 0.05) (Figure 11). It was consistent with the PCR results that EGR1 and PLA2G4A have the higher protein expression in the tissues of renal fibrosis as validated by the Western blot analysis (Figure 12).

## Discussion

Our study analyzed three datasets of gene expression profiles in whole blood, with batch effects removed by the SVA algorithm for subsequent analysis. First, an unsupervised consensus clustering analysis was performed based on the expressions of m6A regulators. The ESTIMATE algorithm evaluated the immune infiltration characteristics in different m6A patterns. Finally, the expression analysis of typical immune checkpoints and immune-related genes was performed. We developed a 5-gene predictive target gene signature based on the WGCNA of drug targets, differential gene analysis, logistic regression analysis, etc. Finally, we performed drug sensitivity and gene and extracellular matrix analysis. Five genes were significantly associated with drug sensitivity and extracellular matrix activity.

m6A is the most prevalent mRNA post-transcriptional methylation in eukaryotic cells (Cao et al., 2016). Especially in mammals, m6A-dependent mRNA modification is a critical process. It regulates multiple biological processes such as self-renewal and differentiation, DNA damage response, tissue development, RNA–protein interactions, and primary microRNA processing by regulating RNA splicing, stability, translocation, and translation into proteins (Cao et al., 2016). Growing literature studies have reported the critical role of m6A methylation in epigenetic regulation and how this modification affects the pathogenesis of various diseases, including renal injury. It has been reported that m6A is involved in the epithelial–mesenchymal transition of cancer cells and is regulated by methyltransferases, demethylases, and m6A-binding proteins (Ma et al., 2021a). Recent evidence suggests that m6A methylation is associated with acute kidney injury (Wang et al., 2019; Xu et al., 2020).

It is known that in the study of key cancer genes, using the gene signature as the input for feature selection may be a better modeling algorithm than genome-wide gene expression profiles when using expression regression models of the lasso strategy (Liu et al., 2021). Traditional genome-wide analysis has been used for an early diagnosis and prognostic model of renal fibrosis (Sun et al., 2022). The reported biomarkers also indicated that renal fibrosis was closely related to immunity, which is consistent with our conclusion. But the correlation with the extracellular matrix is not mentioned in the study. However, we studied the modification pattern based on m6A and found that different m6A modified subtypes have different immune infiltrations. In addition, the key drug targets we found are closely related to the extracellular matrix, indicating the feasibility of our m6A-based gene signature may be more convincing and feasible.

This study revealed that the progression of renal fibrosis is closely related to the m6A methylation pattern. m6A methylation is an abundant endoepigenetic modification that has recently received much attention. Recent studies have shown that m6A modification is essential in regulating immune responses. We divided renal fibrosis patients into three subtypes with different prognoses and different immune statuses based on 21 m6A modulators. Renal fibrosis, a pathological change driven by inflammatory responses and calcium desposits, is a feature of renal transplant failure (Hu et al., 2022). However, it is difficult to characterize the immune status of a specific patient due to the immune heterogeneity in renal fibers. While immune function is broadly regulated by m6A methylation (Li et al., 2021), the three m6A subtypes have enhanced the understanding of the molecular characteristics and subpopulation-specific immune status of renal fibers. These results help predict the clinical treatment outcome of renal fibrosis and search for drug targets. Our follow-up study found significant differences in the expression of immune checkpoints and immune-related genes in different m6A patterns. This means that m6A modification patterns may benefit more from immunotherapy responses. Subsequent analysis of key drug targets based on m6A modified subtypes revealed that key genes were associated with the extracellular matrix, consistent with previous studies on the progression of renal fibrosis (Bülow and Boor, 2019).

EGR1 (Early Growth Response 1) has long been dysregulated in many cancers and is known to regulate tumor progression, making it an attractive target for cancer therapy (Saha et al., 2021). EGR1-activated LINC01503 epigenetically silences the DUSP5/CDKN1A expression, mediating cell cycle progression and tumorigenesis (Su et al., 2019; Ma et al., 2021b). Another example is the loss of Nm23-H1 in invasive breast cancer caused by the downregulation of CTCF and EGR1 (Wong et al., 2021). EGR1 may interact with DNMT3L, inhibit the miR-195-AKT3 axis, and regulate gastric cancer cell apoptosis (Yang et al., 2019). There is a strong correlation between the EGR1 expression and HIV reactivation, with the active transcription in response to the peak expression of EGR1 (Wong et al., 2022). Curcumin sensitizes prolactinoma cells to bromocriptine by activating ERK/EGR1 and inhibiting the AKT/GSK-3β signaling pathway *in vitro* and *in vivo* (Tang et al., 2021). The high expression of EGR1 promotes the proliferation of mast cells, plasma cells, and macrophages, which promote the expansion of the abdominal aorta and affect the immune process (Guo et al., 2022).

The GHR signaling pathways play important roles in growth, metabolism, cell cycle control, immunity, homeostatic processes, and chemoresistance. Dysregulation of GHR signaling is associated with various diseases and chronic diseases, such as acromegaly, cancer, aging, metabolic diseases, fibrosis, inflammation, and autoimmunity (Strous et al., 2020). Wang et al. (2020) showed that GHR gene polymorphisms are associated with esophageal cancer in the general population, and GHR signaling can be applied to cancers and other therapeutic strategies (Gao et al., 2020). The extracellular domain of GHR can be cleaved during shedding, reducing the number of cell-surface signaling receptors, which modulate the sensitivity of cells to GH (Frank, 2001). In muscle tissue, GHR disruption has been reported to enhance insulin sensitivity and prolong lifespan (List et al., 2020).

Phospholipase A2-iva (PLA2G4A) is the most abundant subtype of cytosolic phospholipase A2 (cPLA2) and is an important enzyme in tumorigenesis (Zhang et al., 2018). The eicosanoid signaling pathway based on arachidonic acid (AA) is involved in the development of human cancer. The cytoplasmic phospholipase A2-α (cPLA2α) encoded by the PLA2G4A gene acts as an upstream regulator of the eicosanoid signaling pathway by providing intracellular AA (Bazhan and Khaniani, 2018). Studies have shown that the PLA2G4A gene can be used as a biomarker in various diseases such as gastric cancer (Bazhan and Khaniani, 2018), acute myeloid leukemia (Hassan et al., 2021; Lai et al., 2021), cholangiocarcinoma (Sun et al., 2019), and colorectal cancer (Zhan et al., 2021a). PLA2G4A activates the colorectal cancer microenvironment to produce pro-cytokines IL-17A and adenosine, thereby establishing an effective immunosuppressive microenvironment and promoting immune evasion and tumor metastasis (Zhan et al., 2021b). Trametinib inhibits the cell viability and signaling of organoids to a greater extent by inhibiting the expression of PLA2G4A (Klotsman et al., 2007).

SLC4A1 is a member of the solute carrier family 4 (Zhang et al., 2021b). Studies have shown that this gene is associated with distal renal tubular acidosis (Elhayek et al., 2013; Deejai et al., 2019). The epithelial transporter SLC4A1 is involved in immune response-related biological processes and is characterized by its upregulation in kidney transplantation (Hruba et al., 2019). It is involved in regulatory pathways, including immune response, granulocyte activation, and T cell activation (Han et al., 2021). The SLC4A1-related pathway analysis revealed increased gene enrichment in extracellular matrix–receptor interactions and axon guidance pathways (Saraf et al., 2018). Thymocyte differentiation antigen-1 (THY1) has been reported to affect lung fibroblast proliferation and fibrotic signaling (Chen et al., 2019). In addition, a high expression of Thy1 was associated with poorer recurrence-free survival in breast cancer patients. Thy1 methylation may track the transfer of bipotent progenitors to differentiated cells. Thy1 is a good candidate biomarker for basal-like breast cancer. Thy1 expression was downregulated in xenografts due to promoter methylation. Thy1-knockdown responded to targeted therapy with increased EGFR and Notch1 expressions. THY1 is doxorubicin-resistant in tumors of offsprings exposed to high-fat diets (Montales et al., 2016). Skeletal muscle from patients with type 2 diabetes exhibits degenerative remodeling of the extracellular matrix, which is associated with a selective increase in a subset of fibrolipogenic progenitors marked by the expression of THY1 (Farup et al., 2021). Since DNA methylation is often altered in early cancer development, candidate methylation markers may be used as biomarkers for basal-like breast cancer (Montanari et al., 2019).

## Conclusion

Renal fibrosis is a process of wound-healing failure in renal tissue after chronic injury, calcium deposits, and inflammation. In the study, we used an integrated bioinformatics approach, machine learning, regression algorithms, and *in vitro* experiments to study m6A modifications and drug targets in renal fibrosis. We identified three different m6A subtypes of renal fibrosis through an unsupervised consensus clustering analysis and evaluated immune infiltration characteristics and the expression of immune checkpoints and immune-related genes among distinct m6A subtypes. Overlapping drug targets between WGCNA and DEGs were defined as key drug targets. We used LASSO-SLR to develop a 5 drug target-based prediction model to diagnose renal fibrosis. The 5-gene model had a good predictive effect and was significantly associated with many drugs and extracellular matrix activities. The expression levels of both predictive signature genes EGR1 and PLA2G4A were validated in renal fibrosis and adjacent normal tissues by using the qRT-PCR and Western blot methods.

## Data Availability

The original contributions presented in the study are included in the article/Supplementary Material; further inquiries can be directed to the corresponding authors.

## Supplementary material

SUPPLEMENTARY FIGURE S1 Correlation box plot of M6A_Cluster and Disease_state.

SUPPLEMENTARY TABLE S1 Primers used in PCR.
