# Supplementary material for: Integrated bioinformatical analysis, machine learning and in vitro experiment-identified m6A subtype, and predictive drug target signatures for diagnosing renal fibrosis
Source: Front Pharmacol. 2022 Aug 31;13:909784. doi: 10.3389/fphar.2022.909784 (PMC9470879; doi:10.3389/fphar.2022.909784)
Supplement: Supplementary file 1 [file Table1.DOCX]

| Genes | Sense | Antisense |
| --- | --- | --- |
| EGR1 | 5′-GCAGGCTCGCTCCCACGGTC-3′ | 5′-GGGGTTGGCCGGGTTACATG-3′ |
| PLA2G4A | 5′-AGGTCGACTCTAGAGGATCC-3′ | 5′-TCCTTGTAGTCCATACCCACA-3′ |
| GAPDH | 5′-TGTGGGCATCAATGGATTTGG-3′ | 5′-ACACCATGTATTCCGGGTCAAT-3′ |
