# Supplementary figures and images for: Integrated bioinformatical analysis, machine learning and in vitro experiment-identified m6A subtype, and predictive drug target signatures for diagnosing renal fibrosis
Source: Front Pharmacol. 2022 Aug 31;13:909784. doi: 10.3389/fphar.2022.909784 (PMC9470879; doi:10.3389/fphar.2022.909784)

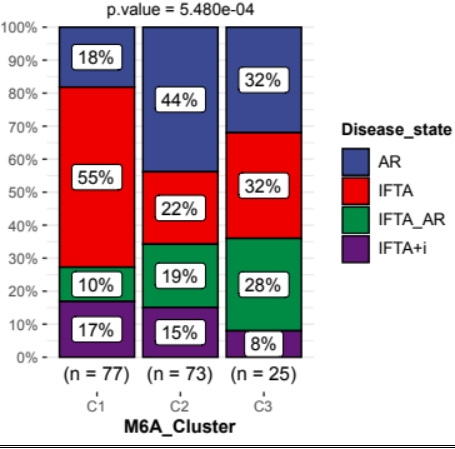

Supplement: Supplementary file 2 [file Image1.JPEG]
